# Comment on: Occurrence of Hookworm and the First Molecular and Morphometric Identification of *Uncinaria stenocephala* in Dogs in Central Europe

**DOI:** 10.1007/s11686-023-00737-3

**Published:** 2023-11-22

**Authors:** Marta Demkowska-Kutrzepa, Klaudiusz Szczepaniak, Monika Roczeń-Karczmarz, Maria Studzińska, Krzysztof Tomczuk

**Affiliations:** Department of Parasitology and Fish Diseases, University of Life Sciences in Lublin, Lublin, Poland

**Keywords:** Hookworms, *Uncinaria stenocephala*, Molecular analysis

## Abstract

**Background:**

Hookworms are blood-feeding nematodes that infect millions of people and animals worldwide. The most common species infecting dogs in Europe are representatives of the family Ancylostomatidae, which differ in invasiology, biology and morphological features. However, the differentiation of invasions of species such as *Uncinaria stenocephala* and *Ancylostoma caninum* based on a coproscopic examination is problematic. For this reason, it is recommended to use molecular diagnostics for this purpose. The authors of the article investigated the prevalence of *U. stenocephala* in dogs kept in various living conditions in Slovakia and developed a two-step morphology–molecular analysis-based strategy to identify the genus and the species of eggs and larvae of the Ancylostomatidae family in dogs.

**Conclusions:**

In our opinion, this work is very much needed as it shows how to effectively diagnose hookworm infestations. However, we do not agree with the information in the title of the article because such studies have already been carried out in Central Europe by other authors.

We commend the contribution of the study; however, the title of the article suggests that the described research was the first in Central Europe morphometric and molecular identification of the species *U. stenocephala*. We absolutely disagree with it.

In 2018, we published an article on the morphometric and molecular identification of the species *U. stenocephala* [1]. In the publication, we presented both the analysis of morphometric features of eggs and molecular studies. Similar research extended with morphometric evaluation of hookworm larvae was performed in Slovakia in 2022 by Štrkolcová* et al*. but after reviewing the European literature, it should not be concluded that authors made a morphometric and molecular analysis of *U. stenocephala* as the first in Central Europe.

Additionally, to correct the citation, we wanted to provide the correct data of our article in the reference list.
